# Psychotropic drugs and the risk of fractures in old age: a prospective population-based study

**DOI:** 10.1186/1471-2458-10-396

**Published:** 2010-07-06

**Authors:** Janne Nurminen, Juha Puustinen, Maarit Piirtola, Tero Vahlberg, Sirkka-Liisa Kivelä

**Affiliations:** 1 Family Medicine, Institute of Clinical Medicine, University of Turku, Lemminkäisenkatu 1, Turku, 20014, Finland; 2 Härkätie Health Center, PL 51, Lieto, 21421, Finland, and Turku Health Center, PL 670, Turku, 20101, Finland; 3 Unit of Neurology, Satakunta Central Hospital, Sairaalantie 3, Pori, 28500, Finland; 4 Department of Biostatistics, Institute of Clinical Medicine, University of Turku, Lemminkäisenkatu 1, Turku, 20014, Finland; 5 Turku University Hospital, PL 52, Turku, 20521, Finland, and Satakunta Central Hospital, Sairaalantie 3, Pori, 28500, Finland

## Abstract

**Background:**

There is evidence that the use of any psychotropic and the concomitant use of two or more benzodiazepines are related to an increased risk of fractures in old age. However, also controversial results exist. The aim was to describe associations between the use of a psychotropic drug, or the concomitant use of two or more of these drugs and the risk of fractures in a population aged 65 years or over.

**Methods:**

This study was a part of a prospective longitudinal population-based study carried out in the municipality of Lieto, South-Western Finland. The objective was to describe gender-specific associations between the use of one psychotropic drug [benzodiazepine (BZD), antipsychotic (AP) or antidepressant (AD)] or the concomitant use of two or more psychotropic drugs and the risk of fractures in a population 65 years or over. Subjects were participants in the first wave of the Lieto study in 1990-1991, and they were followed up until the end of 1996. Information about fractures confirmed with radiology reports in 1,177 subjects (482 men and 695 women) during the follow-up was collected from medical records. Two follow-up periods (three and six years) were used, and previously found risk factors of fractures were adjusted as confounding factors separately for men and women. The Poisson regression model was used in the analyses.

**Results:**

The concomitant use of two or more BZDs and the concomitant use of two or more APs were related to an increased risk of fractures during both follow-up periods after adjusting for confounding factors in men. No similar associations were found in women.

**Conclusions:**

The concomitant use of several BZDs and that of several APs are associated with an increase in the risk of fractures in older men. Our findings show only risk relations. We cannot draw the conclusion that these drug combinations are causes of fractures.

## Background

Fractures in old age create a major health problem with considerable disability and mortality. Fractures are typically caused by a combination of a fall and an underlying bone disease, e.g., osteoporosis [1]. Several risk factors for falls and fractures have been identified, of which the use of psychotropics, i.e., benzodiazepines (BZDs), antidepressants (ADs) or antipsychotics (APs), belongs to avoidable ones [2]. The use of any psychotropic was found to be a risk factor of fractures in a South-Korean cohort study [3].

Large cohort studies have shown that the use of BZDs increases the risk of fractures in the aged [4-6]. There are, however, also controversial results [7]. In the majority of case-control studies, positive relationships between BZD, hypnotic or sedative use and the risk of fractures have been found [8-10], while some studies present no such findings [11]. Two surveys have revealed that the concomitant use of BZDs raises the risk of fractures up to 2.5-fold compared with the non-use [8,12].

According to case-control and cohort studies, the use of ADs increases the risk of fractures in old age [13-16]. Cohort studies concerning the use of APs and the risk of fractures are rare. Jacqmin-Gadda *et al. *(1998) did not find a positive association between the use of APs and the risk of fractures [15]. However, positive associations from case-control studies exist [9,17,18].

The concomitant use of psychotropics is common in the aged [19]. Despite this, associations between the concomitant use of psychotropics and the risk of fractures have not been studied in detail. The concomitant use may create a stronger risk than the use of one psychotropic alone [8]. According to our knowledge, our prospective population-based epidemiological study is the first cohort study to test the hypothesis about the associations between the concomitant use of psychotropics and the risk of fractures in the aged.

The aim was to describe associations between the use of a benzodiazepine or a related drug (BZD), an antipsychotic (AP), an antidepressant (AD), or the concomitant use of two or more of these drugs and the risk of fractures in a population aged 65 years or over.

## Methods

### Study population and examinations

This study was a part of a larger longitudinal, unselected, population-based study carried out in the municipality of Lieto, South-Western Finland [20]. The baseline data was collected between October 1, 1990, and December 31, 1991, and the population consisted of all the residents in Lieto born in 1926 or earlier (n = 1,283). Of these residents, 1,196 (93%), 488 men and 708 women, participated in the baseline study.

The methods and variables used have been described in detail in previous reports [20,21].

### Data on fractures

Information about fractures confirmed with radiology reports was collected individually from the medical records from the baseline until the end of 1996. Data on fractures were obtained for 1,177 participants (482 men and 695 women) (98% of the baseline population), who formed the subjects of this study [21].

Only the first fracture of each participant during the follow-up period was included. Pathological fractures and those caused by the most serious accidents were excluded [20,21]. In the case of persons who sustained more than one fracture in an accident, the main fracture contributing to the need for treatment was taken into account. Fractures were classified using ICD-10 codes.

### Medication data

Participants were asked to take their prescription forms, medication lists and pill boxes to the examinations. Data on the use of drugs were collected by a trained nurse by interviewing and by checking the prescription forms, medication lists, pill boxes and medical records of the participants.

Drugs were defined by using the Anatomical Therapeutic Chemical (ATC) Classification 2000 [22]. The groups of drugs defined as those with effect on central nervous system (CNS) and used in the analyses were as follows: *opioids (OPs) *(ATC code N01AH, N02A, N02BE51, R05DA, R05FA), *antiepileptic drugs (AEs) *(ATC code N03A), *anticholinergic drugs (ACHs) *(ATC codes N04A, N05AA01, N05AA02, N05AB01, N05AB02, N05AB03, N05AB04, N05AC01, N05AC02, N05AF01, N05AF03, N05AF05, N05BB01, N06AA04, N06AA06, N06AA09, N06AA12, N02AG, A03AA, A03AB, A03AX03, A03B, A03CA, A03CB31, A03DA, A03FA01, A04AD01, A04AD12, C01BA01, C01BA03, C01BA51, C01BA71, R03BB, M03B, G04BD, S01FA, R01BA01, R01BA51, R06AB01, R06AE03, R06AE53), *benzodiazepines and related drugs (BZDs) *(ATC codes N05BA, N05CD, N03AE01, N05CF, A03CA, C01DA70, M05AA51, N06CA01, N02BA71), *antipsychotics (APs) *(ATC codes N05A, N06CA01) and *antidepressants (ADs) *(ATC codes N06A, N06CA). Antipsychotics, antidepressants and benzodiazepines together were classified as psychotropic drugs.

### Confounding variables

The selection of confounding variables was based on the results of the previous study describing the predictors of fractures within the same study population [21]. According to these results, high age, lowered handgrip strength (<76 kPa), body mass index (BMI) under 30 kg/m^2^, and a compression fracture in one or more upper lumbar or thoracic vertebras are independent risk factors of fractures in women, whereas high age, great number of depressive symptoms, and a compression fracture in one or more upper lumbar or thoracic vertebras were risk factors in men [21]. These predictors of fractures were used as confounding variables in gender-specific analyses in this study.

The weight (in light clothes) and height of the participants were measured in order to define body mass index (BMI) [body mass (kg) divided by height (meters) squared]. Depressive symptoms were assessed with the Zung Self-rating Depression Scale (ZSDS). Handgrip strength, as an indicator of muscle strength, was measured with an Elmed Vigorimeter^® ^(hand dynamometer). Chest X-rays were taken at baseline from two different angles and were examined by the same experienced general practitioner. A compressed fracture was diagnosed if a vertebra was obviously compressed to a wedge shape. Only definite compression fractures were recorded.

### Statistical analyses

The differences in the use of drugs between men and women were compared with chi-square test, or Fisher's exact test. In order to describe associations between the use of drugs and the risk of fractures, two follow-up periods (three and six years) were used. Subjects were followed up from the baseline (October 1, 1990) to the occurrence of the first fracture, and subjects with no fractures were followed up to the end of the follow-up period (December 31, 1993, or December 31, 1996) or to death.

The associations between a certain group of drugs or combinations of drugs and fractures were analyzed at the first phase using univariate and age-adjusted Poisson regression analyses. Participants who did not use any psychotropic or other drug affecting the central nervous system (opioid, anticholinergic or antiepileptic medication) were used as a control group. In the second phase, drugs or combinations of drugs showing significant associations with fractures in age-adjusted analyses were adjusted for confounding variables. Results were quantified using relative risks (RR) with their 95% confidence intervals (CI). Analyses were done separately for men and women. P-values less than 0.05 were considered statistically significant. Statistical analyses were carried out by using SAS System for Windows version 9.1 (SAS Institute Inc., Cary, NC).

### Ethics approval and patient consent

Informed consent was obtained from all participants or their caregivers. The baseline plan was approved by the Joint Ethics Committee of the University of Turku and the Turku University Central Hospital. Data collection for follow-up was approved by the Finnish Ministry of Social Affairs and Health, the Finnish National Research and Development Centre for Welfare and Health, and the Lieto District Health Authority.

## Results

### Participant characteristics

Nearly all participants lived in their own homes and were able to walk independently (Table 1). On average, they used 2.6 prescribed drugs regularly and 0.6 prescribed drugs irregularly. The concomitant use of several psychotropics was not common (Table 2).

**Table 1:** Participant characteristics, N = 1,177

	No. (%)
**Sex**	

Women	695 (59.0)

Men	482 (41.0)

**Marital status**	

Married	649 (55.1)

Unmarried or divorced	131 (11.1)

Widowed	397 (33.7)

**Place of living**	

Home, with other person(s)	749 (63.6)

Home, alone	364 (30.9)

Institution	64 (5.4)

**Basic education**	

Less than basic	118 (10.0)

Basic	979 (83.2)

More than basic	80 (6.8)

**Walking ability**	

Independently	956 (81.5)

Using a device	167 (14.2)

With help of other person(s)	50 (4.3)

**Previous occupation**	

Service	205 (17.4)

Industry	421 (35.8)

Agriculture	453 (38.5)

Family	98 (8.3)

**Smoking**	

Current	94 (8.0)

Former	303 (25.8)

	**Mean ± SD**

**Age, yrs**	73.2 ± 6.9

**Body mass index, kg/m**^**2**^	27.7 ± 4.9

**Number of drugs**	

Regular	2.6 ± 2.5

Irregular	0.6 ± 1.0

**Table 2:** Use and concomitant use of psychotropics at baseline among 1,177 participants

Drug	Men (n = 482)	Women (n = 695)	**P-value**^**a**^
		
	No. (%)	No. (%)	
One BZD	76 (15.8)	187 (26.9)	<.001

One AP	31 (6.4)	64 (9.2)	.10

One AD	14 (2.9)	38 (5.5)	.04

At least two BZDs	13 (2.7)	37 (5.3)	.03

At least two APs	5 (1.0)	9 (1.3)	.79

At least two ADs	1 (0.2)	0 (0.0)	.41

BZD and AP^b^	19 (3.9)	29 (4.2)	.88

BZD and AD^b^	10 (2.1)	25 (3.6)	.16

AP and AD^b^	5 (1.0)	13 (1.9)	.34

At least one psychotropic drug	92 (19.1)	229 (32.9)	<.001

a statistical difference between the sexes

b persons using concomitantly one drug or more drugs from each subgroup

**BZD **= benzodiazepine or related drug; **AP **= antipsychotic drug; **AD **= antidepressant

### Fractures and deaths

During the follow-up of three years (10/01/1990-12/31/1993), 113 participants (9.6% of the baseline population), 29 men and 84 women, sustained 121 fractures (Table 3). In the follow-up of six years (10/01/1990-12/31/1996), 178 participants (15.1% of the baseline population), 45 men and 133 women, sustained 221 fractures. Altogether, 160 participants died during the three-year follow-up period, and 312 participants during six years.

**Table 3:** Distribution of fracture types during the follow-up periods of three and six years among 1,177 participants taking into account the first fracture

Type of fracture	Men (n = 482)		Women (n = 695)	
	
	Three years	Six years	Three years	Six years
	No. (%)	No. (%)	No. (%)	No. (%)
Total	29 (100)	45 (100)	84 (100)	133 (100)

Hip	6 (20.7)	8 (17.8)	17 (20.2)	29 (21.8)

Wrist	3 (10.3)	5 (11.1)	25 (29.8)	40 (30.1)

Tibial and ankle	7 (24.1)	9 (20.0)	10 (11.9)	12 (9.0)

Prox. humerus	1 (3.4)	2 (4.4)	10 (11.9)	15 (11.3)

Rib(s)	2 (6.9)	9 (20.0)	3 (3.6)	7 (5.3)

Vertebral compression	3 (10.3)	3 (6.7)	6 (7.1)	8 (6.0)

Other	7 (24.1)	9 (20.0)	13 (15.5)	22 (16.5)

### Drugs and the risk of fractures

In univariate analyses, the use of two or more BZDs was associated with an increased risk of fractures in men during both follow-up periods (Table 4). These relationships remained significant after adjusting for confounding variables. Poisson regression analysis adjusted for confounding variables also showed the use of two or more APs to be associated with an increased risk of fractures in men during both follow-up periods.

**Table 4:** Relationships between use of psychotropics and risk of fractures in men

Drug	Number of fracture cases No. (%)/controls No. (%)	Univariate analysis		Age-adjusted analysis		**Adjusted analysis**^**a**^	
		
		RR (95% CI)	P-value	RR (95% CI)	P-value	RR (95% CI)	P-value
**3-year follow-up**							

One BZD	7 (9.2)/20 (5.7)	1.7 (0.7-4.1)	.21	1.4 (0.6-3.4)	.44	-	-

One AP	2 (6.5)/20 (5.7)	1.3 (0.3-5.4)	.76	0.9 (0.2-4.1)	.93	-	-

One AD	1 (7.1)/20 (5.7)	1.3 (0.2-9.5)	.81	1.0 (0.1-7.9)	.97	-	-

Two or more BZDs	3 (23.1)/20 (5.7)	4.9 (1.4-16.4)	.01	4.7 (1.4-15.7)	.01	4.7 (1.4-16.3)	.01

Two or more APs	1 (20.0)/20 (5.7)	7.0 (0.9-52.0)	.06	9.4 (1.2-71.1)	.03	8.3 (1.0-66.2)	.05

Two or more ADs	0 (0.0)/20 (5.7)	-	-	-	-	-	-

BZD and AP^b^	2 (10.5)/20 (5.7)	2.3 (0.5 - 9.8)	.27	2.5 (0.8-8.4)	.13	-	-

BZD and AD^b^	1 (10.0)/20 (5.7)	1.9 (0.3-14.2)	.53	1.5 (0.2-11.3)	.67	-	-

AP and AD^b^	1 (20.0)/20 (5.7)	4.9 (0.7-36.6)	.12	4.9 (0.7-36.0)	.12	-	-

**6-year follow-up**							

One BZD	11 (14.5)/29 (8.3)	2.1 (1.1-4.3)	.03	1.8 (0.9-3.7)	.09	-	-

One AP	4 (12.9)/29 (8.3)	2.0 (0.7-5.6)	.20	1.6 (0.6-4.8)	.37	-	-

One AD	1 (7.1)/29 (8.3)	1.0 (0.1-7.1)	.98	0.9 (0.1-6.6)	.91	-	-

Two or more BZDs	4 (30.8)/29 (8.3)	5.5 (1.9-15.8)	.001	5.7 (2.0-16.2)	.001	5.8 (2.0-16.6)	.001

Two or more APs	1 (20.0)/29 (8.3)	6.2 (0.8-45.5)	.07	7.6 (1.0-56.7)	.05	7.9 (1.1-59.0)	.04

Two or more ADs	0 (0.0)/29 (8.3)	-	-	-	-	-	-

BZD and AP^b^	3 (15.8)/29 (8.3)	2.8 (0.7-9.3)	.09	2.5 (0.8-8.4)	.13	-	-

BZD and AD^b^	1 (10.0)/29 (8.3)	1.6 (0.2-11.8)	.64	1.5 (0.2-11.3)	.67	-	-

AP and AD^b^	1 (20.0)/29 (8.3)	4.8 (0.7-35.5)	.12	4.9 (0.7-36.0)	.12	-	-

a adjusted for age, amount of depressive symptoms in Zung Self-rating Depression Scale and a compression fracture in one or more upper lumbar or thoracic vertebras at baseline

b persons using concomitantly one drug or more drugs from each subgroup

**BZD **= benzodiazepine derivative or related drug; **AP **= antipsychotic drug; **AD **= antidepressant; **P-value **= statistical significance; **RR **= relative risk; **CI **= confidence interval

In women, the use of two or more APs was related to an increased risk of fractures in a univariate analysis in the three-year follow-up, but this relationship did not remain significant after adjusting for age (Table 5).

**Table 5:** Relationships between use of psychotropics and risk of fractures in women

Drug	Number of fracture cases No. (%)/controls No. (%)	Univariate analysis		Age-adjusted analysis		**Adjusted analysis**^**a**^	
		
		RR (95% CI)	P-value	RR (95% CI)	*P *Value	RR (95% CI)	P-value
**3-year follow-up**							

One BZD	22 (11.8)/46 (11.9)	1.0 (0.6-1.7)	.88	0.9 (0.5-1.5)	.66	-	-

One AP	9 (14.1)/46 (11.9)	1.3 (0.6-2.7)	.47	1.1 (0.5-2.2)	.83	-	-

One AD	3 (7.9)/46 (11.9)	0.7 (0.2-2.2)	.52	0.6 (0.2-1.9)	.36	-	-

Two or more BZDs	6 (16.2)/46 (11.9)	1.4 (0.6-3.4)	.40	1.3 (0.5-3.0)	.60	-	-

Two or more APs	3 (33.3)/46 (11.9)	3.8 (1.2-12.2)	.03	3.3 (1.0-10.7)	.05	1.0 (0.1-7.0)	.98

Two or more ADs	0 (0.0)/46 (11.9)	-	-	-	-	-	-

BZD and AP^b^	5 (17.2)/46 (11.9)	1.7 (0.7-4.4)	.24	1.4 (0.6-3.6)	.46	-	-

BZD and AD^b^	2 (8.0)/46 (11.9)	0.7 (0.2-2.9)	.62	0.6 (0.1-2.5)	.47	-	-

AP and AD^b^	2 (15.4)/46 (11.9)	1.4 (0.3-5.7)	.65	1.1 (0.3-4.7)	.86	-	-

**6-year follow-up**							

One BZD	33 (17.7)/76 (19.6)	1.0 (0.6-1.5)	.89	0.8 (0.6-1.3)	.43	-	-

One AP	13 (20.3)/76 (19.6)	1.2 (0.7-2.2)	.54	1.0 (0.6-1.8)	.98	-	-

One AD	5 (13.2)/76 (19.6)	0.7 (0.3-1.8)	.48	0.6 (0.2-1.5)	.30	-	-

Two or more BZDs	7 (18.9)/76 (19.6)	1.0 (0.5-2.2)	.95	0.9 (0.4-2.0)	.81	-	-

Two or more APs	3 (33.3)/76 (19.6)	2.9 (0.9-9.2)	.07	2.6 (0.8-8.3)	.10	-	-

Two or more ADs	0 (0.0)/76 (19.6)	-	-	-	-	-	-

BZD and AP^b^	7 (24.1)/76 (19.6)	1.6 (0.8-3.6)	.21	1.4 (0.6-3.0)	.43	-	-

BZD and AD^b^	3 (12.0)/76 (19.6)	0.7 (0.2-2.1)	.48	0.6 (0.2-1.8)	.34	-	-

AP and AD^b^	4 (30.8)/76 (19.6)	1.9 (0.7-5.2)	.21	1.6 (0.6-4.4)	.38	-	-

a Adjusted for age, hand grip strength, body mass index (BMI) and a compression fracture in one or more upper lumbar or thoracic vertebras at baseline

b Persons using concomitantly one drug or more drugs from each subgroup

**BZD **= benzodiazepine derivative or related drug; **AP **= antipsychotic; **AD **= antidepressant; **P-value **= statistical significance; **RR **= relative risk; **CI **= confidence interval

## Discussion

Our results show that the concomitant use of two or more BZDs and the concomitant use of two or more APs are related to an increased risk of fractures in older men. These findings were evident after adjusting for confounding variables in the follow-up periods of either three or six years.

Our study is a longitudinal prospective population-based cohort study with accurate data about fractures during the follow-up periods, use of drugs at baseline and other baseline variables. These are the major methodological strengths. Many studies concerning associations between the use of psychotropics and the risk of fractures are based on a retrospective case-control setting that has a limited ability to reveal causal relations [8,13,14,17]. We were able to adjust our data for risk factors of fractures. These confounding variables were found in the previous study from the same data [21]. However, only risk relations can be estimated in an epidemiological prospective follow-up study, and the associations found here do not show causalities.

All fractures were confirmed by radiologists, and the use of drugs was confirmed from prescription forms, pill boxes and medical records to minimize recall bias.

Even though the numbers of fractures during the follow-up periods (113 in three and 178 in six years) were rather high, the number did not allow analyses by types of fractures. The numbers of participants using a certain drug or a combination of drugs were not high for every drug. This possesses statistical limitations. Confidence intervals tended to be wide, and there is a lack of statistical power in many Poisson regression model analyses.

In general, controlling confounding factors has posed problems in many previous case-control studies [23,24]. Many previous studies have used administrative databases, which do not contain the valuable information about potential confounders [8,13]. The results of hospital-based case-control studies may be unreliable because of possible control selection bias [24].

The only muscle strength test included in the Lieto study was the handgrip strength test. The handgrip strength was measured three times in a row with a hand dynamometer. The average value of these three values was calculated and recorded. The handgrip strength was used as an overall muscle strength approximation because of its feasibility in this population-based study. There is evidence that handgrip strength reflects overall muscle strength and self-rated health [25]. In addition, lowered handgrip strength is an independent predictor of hospitalization [26] and excess mortality [27]. Despite of this, the use of specific muscle strength tests for lower extremities may have given more reliable results concerning participants' muscle strength of lower extremities.

All certain vertebral compression fractures found in chest X-rays were recorded during the baseline data collection. Routine chest X-rays were not performed during the follow-up, but all vertebral compression fractures diagnosed in clinical examinations during the follow-up have been recorded (Table 3). Vertebral compression fracture may not cause any clinical symptoms, or the symptoms may be so mild that the patient does not take contact with a doctor. Because of this, we may have missed a group of undiagnosed vertebral compression fractures. This must be taken into account when assessing the results.

Our control group consisted of persons not using any psychotropic, opioid, antiepileptic or anticholinergic drugs. In addition to psychotropics, there is evidence that opioids and antiepileptics may be related to a high risk of falling and fractures [1,28]. Anticholinergic medications can cause side-effects affecting the central nervous system, and are related to the risk of cognitive decline [29], and may, thus, raise the risk of falls. Our control group was formed in order to compare the users of psychotropic(s) with persons using no drug with known effects on the central nervous system.

Another limitation is that the data on use of drugs was collected only at baseline. It is possible that the use of psychotropics changed during the follow-up periods at least among some participants. The two cross-sectional studies in Lieto in 1990-1991 and 1998-1999 showed that the proportions of users of psychotropics were similar in both waves [30]. This finding supports the hypothesis of the long term use of these drugs. We suggest that the use of psychotropic drugs was long-lasting among many participants.

Our results may be generalized to a population of 65 years or over living in a semi-industrialized municipality [21]. Although the age of the participants has been limited to 65 years or over in the majority of previous studies, many surveys have also included younger participants [10,31]. Therefore, the results of such studies are not entirely comparable with those of ours. The subjects of many previous cohort surveys have only comprised women [7,32]. Many studies have focused on hip fractures, and their results cannot be generalized to all fractures [6,33].

One problem in comparing our results with the previous studies lies in the different definitions of drugs. Many previous studies have used the definitions "hypnotics" or "sedatives", but no exact descriptions about these groups have been written [15,32]. In some surveys, specific drug preparations, doses or both have been analyzed separately, which was not an objective in this study.

Previous cohort studies that have found a positive association between the use of BZDs and the risk of foot fractures have reported the risk ratio to be up to 1.72 [5]. Jacqmin-Gadda *et al. *(1998) found that the use of an anxiolytic creates a 2.25-fold risk for a hip fracture [15]. We did not find a significant association between the use of one BZD and the risk of fractures. The differences in defining the fractures may partly explain the variation in results.

The risk ratio between the concomitant use of two or more BZDs and the risk of fractures has been found to be 2.10 [12]. A case-control study showed that the concomitant use of two or more BZDs creates a 2.5-fold risk for femur fracture [8]. We found the risk ratio for any kind of fracture to be 4.9 in men using two or more BZDs. Our results suggest that the concomitant use of several BZDs increases the risk of fractures in men.

We did not find a positive association between the use of one AD and the risk of fractures, whereas Spector *et al. *(2007) found a 1.5-fold risk for the use of AD in a nursing home setting [16]. The difference in the setting may explain the different results [16]. Ensrud *et al. *(2003) found a significant association between the use of AD and hip fractures in community-dwelling older women [7]. The study was targeted to hip fractures in women, which may partially explain the difference with our results. No previous studies about the concomitant use of ADs and the risk of fractures were found in our literature search. The concomitant use of two ADs is rare in clinical practice. It may lead to a potentially lethal serotonin syndrome, and should, thus, be avoided. No users of two or more ADs were found in our material.

The use of two or more APs was found to be related to an increased risk of fractures in men during both follow-up periods. The relationship between the concomitant use of APs and the risk of fractures has not been analyzed in previous studies. The use of one AP has been found to be associated with an increased risk of fractures in case-control studies [17,18]. The only cohort study did not show a positive association [15].

In our study, the results were similar for both follow-up periods. The results indicate that the associations between the concomitant use of BZDs or that of APs and the risk of fractures remain constant during the years of usage, assuming the drug use remained constant during the follow-up years. According to a previous review, the use of one BZD or that of one AP is associated with the risk for falls in older populations [2]. The concomitant use of several BZDs or APs may have more harmful effects on postural balance than the use of only one drug. Persons using several BZDs or APs may suffer more from problems in postural balance or sedation caused by the excessive use of drugs. These side-effects may increase the risk for falls and fractures. Underlying diseases causing the concomitant use of these drugs may also have a negative effect on physical functioning. Other underlying factors, still undetectable, may exist as well, such as straight negative effects of drugs on bone quality.

Why was the positive association found only in men? More women used benzodiazepines and psychotropics compared to men. Men and women used similar psychotropic preparations in equal doses. Triazolam, oxazepam and lorazepam were the most common BZDs in both sexes. Levomepromazine and perphenazine were the most common antipsychotics, and amitriptyline and doxepine the most common antidepressants in both men and women. The differences between the sexes cannot be explained by the types or doses of the psychotropics. One explanation may be the greater tendency of women to fall and to sustain fractures despite the use of psychotropics. Other risk factors than the use of psychotropics, e.g. poor muscle strengths, may play a greater role in women.

No man using two or more BZDs or APs used per oral corticosteroids suggesting that the increase in fracture risk in these groups was not due to use of per oral corticosteroid.

Another explanation could be that the burden of chronic diseases was heavier in men than in women. This would suggest that at least a part of the identified increase in fracture risk in men was associated with diseases treated with psychotropics.

There is evidence that lower urinary tract symptoms increase the risk of falls in older men [34]. However, also urinary incontinence, that is common in women, has been identified as one of the risk factors for falls [35]. These symptoms may increase the risk of falls by forcing the participants to use the toilet at night time when they are sleepy and the light may be poor. According to the literature, it is difficult to conclude if the symptoms of benign prostate hyperplasia, for example, create a greater risk of falling in men than the symptoms of urinary incontinence in women.

Information concerning participants' alcohol intake and bone mineral density did not belong to the baseline variables. Finnish older men consume more alcohol than women, but the consumption decreases with age [36]. There is, thus, a possibility that the concomitant use of alcohol with two or more BZDs or APs has exposed men to a fall (and a fracture). Osteoporosis is more common in women than in men, but it is also known that diagnosing and treating osteoporosis in men are inadequate [37].

## Conclusions

The hypothesis, if there is a relationship between the concomitant use of several psychotropic drugs and the risk of fracture, has not been studied previously. We tested this hypothesis, and our results show that the concomitant use of two or more BZDs and the concomitant use of two or more APs are related to an increased risk of fractures in older men. These findings were evident after adjusting for confounding variables. Our findings show only risk relations. We cannot draw the conclusion that these drug combinations are causes of fractures.

## Competing interests

The authors declare that they have no competing interests.

## Authors' contributions

JN is the primary responsible for designing, analyzing and writing the article and the data. S-LK has directed and supervised the writing and analyzing process. JP has been planning the data analyses and has contributed to the writing process. MP has collected the fracture data and has significantly contributed to the analyses and the writing process. TV has planned and supervised the biostatistical analyses and has been the advisor of the writing process from statistical point of view. All authors have read and approved the final manuscript.

## Pre-publication history

The pre-publication history for this paper can be accessed here:

http://www.biomedcentral.com/1471-2458/10/396/prepub
